# Immune checkpoint inhibitors and acute kidney injury

**DOI:** 10.3389/fimmu.2024.1353339

**Published:** 2024-02-23

**Authors:** Ping Zhou, Ying Gao, Zhijuan Kong, Junlin Wang, Shuxuan Si, Wei Han, Jie Li, Zhimei Lv, Rong Wang

**Affiliations:** ^1^ Department of Nephrology, Shandong Provincial Hospital, Shandong University, Jinan, China; ^2^ Department of Nephrology, Shandong Provincial Hospital Affiliated to Shandong First Medical University, Jinan, China

**Keywords:** acute kidney injury, nephrotoxicity, immune checkpoint inhibitors, immunotherapy, immune-related adverse events, malignancies

## Abstract

As a new type of anti-tumor immunotherapy, immune checkpoint inhibitors (ICIs) have improved the prognosis of multiple malignancies. However, renal complications are becoming more frequent. Nephrotoxicity often manifests as acute kidney injury (AKI), and the most common histopathological type is acute tubulointerstitial nephritis (ATIN). Based on previous studies of the incidence and potential risk factors for nephrotoxicity, in this review, we describe the mechanism of AKI after ICIs treatment, summarize the incidence, risk factors, and outcomes of AKI, and discuss the diagnosis and management of immune checkpoint inhibitors-associated acute kidney injury (ICI-AKI). In addition, we review the current status of ICIs rechallenge and the therapeutic strategies of ICIs applied in kidney transplant recipients. Finally, we emphasize the importance of collaboration between nephrologists and oncologists to guide the treatment of ICIs and the management of renal complications.

## 1 Introduction

Immune checkpoint inhibitors (ICIs), a kind of monoclonal antibodies, have been confirmed to significantly improve the overall prognosis of a wide range of malignancies (1–3). However, ICIs also lead to the loss of peripheral tolerance of autoantigens, which leads to autoimmune reactions, called immune-related adverse events (irAEs). The most commonly involved organs and systems include gastrointestinal tract, endocrine, skin, etc (4, 5). Similarly, the nephrotoxicity of ICIs has attracted more and more attention. It usually presents as acute kidney injury (AKI), which can cause irreversible loss of renal function in severe cases (6), and needs to be paid attention to.

In this review, we describe the therapeutic mechanism of ICIs and the possible mechanism leading to AKI, summarize the incidence, risk factors, other clinical characteristics and outcomes of AKI after ICIs treatment, discuss the diagnosis and management of immune checkpoint inhibitor–associated AKI, and focus on the application of ICIs for anti-tumor therapy in kidney transplant recipients.

## 2 Development of immune-related adverse events

**Figure 1:**
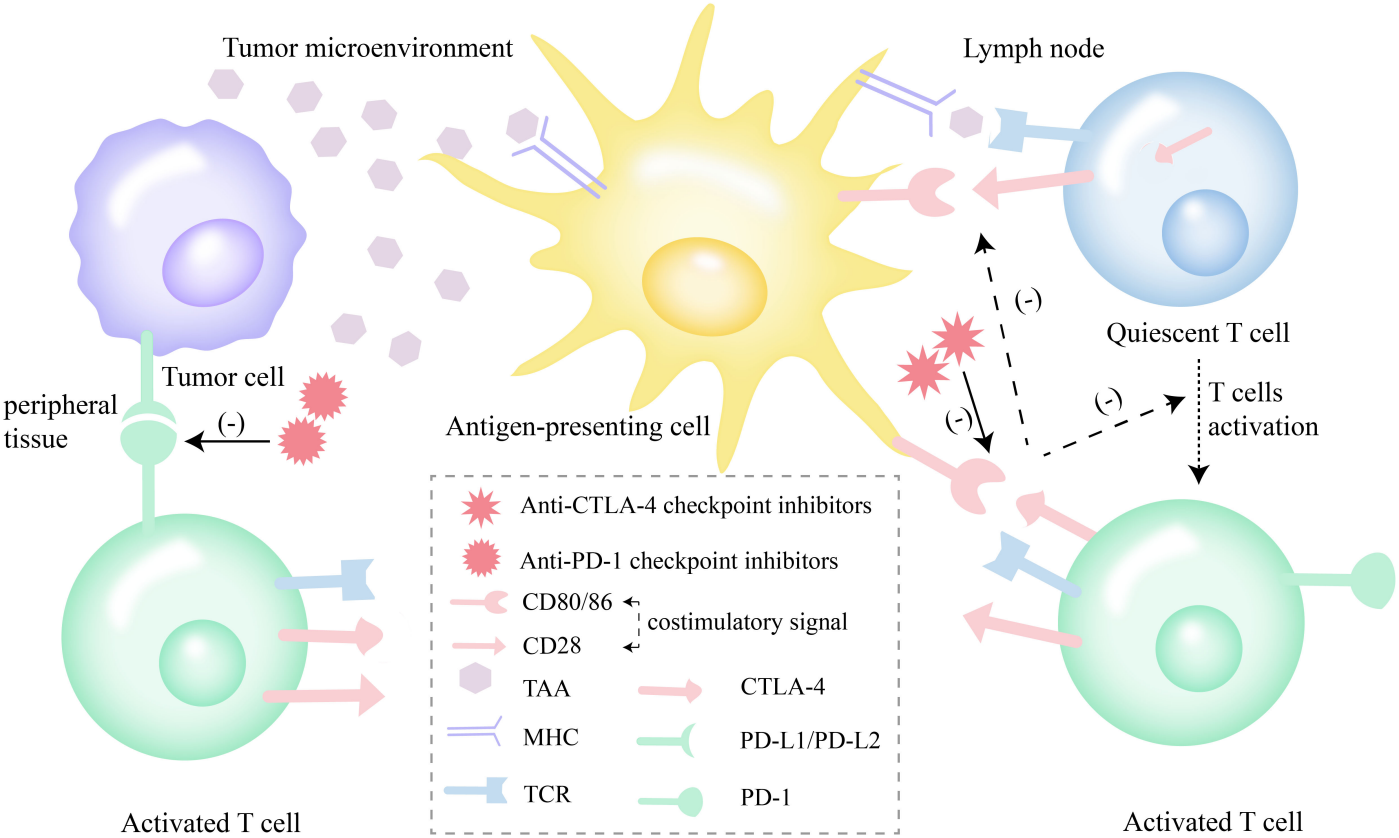
Effect of ICIs (anti-CTLA-4 and anti-PD-1) on T-cell function. Anti-CTLA-4 checkpoint inhibitors enhance anti-tumor response by promoting the activation of quiescent T cells in lymph nodes. Anti-PD-1 checkpoint inhibitors impede the exhaustion of activated T cells by blocking of the PD-1 axis. ICIs, immune checkpoint inhibitors; PD-1, programmed cell death receptor-1; PD-L1, programmed cell death ligand-1; PD-L2, programmed cell death ligand-2; CTLA-4, cytotoxic T lymphocyte-associated antigen-4; TAA, tumor-associated antigens; MHC, major histocompatibility complex; TCR, T cell receptor.

Immune checkpoint inhibitors can not only improve the activity of immune system and enhance the anti-tumor immune response of the body (**Figure 1**), but also cause the loss of peripheral tolerance of autoantigens, resulting in autoimmune reactions, which are recognized as immune-related adverse events (irAEs). The pathogenesis of irAEs includes inflammatory response caused by cytokines, cross-reaction of similar antigens and complement-mediated direct damage (7). The occurrence of irAEs is affected by many factors. For example, autoimmune tendency, body mass index (BMI), and the treatment regimen of ICIs are related to the incidence of irAEs (7). and tumor types is related to the type of irAEs (7–9).

Multiple different systems have been reported to develop irAEs, involving endocrine, gastrointestinal, skin, musculoskeletal, urinary and other systems (7, 10). In particular, attention should be paid to the neurological and fatal irAEs. timely detection and intervention to prevent further deterioration and irreversible damage (11). Currently, treatment strategies for irAEs include screening for autoantibodies in patients who are predisposed to autoimmunity before ICIs are administered, grading treatment and deciding whether to discontinue ICIs according to the severity of irAEs (7, 12). The treatment of irAEs includes corticosteroids, immunosuppressive agents, intravenous immunoglobulin, monoclonal antibodies, and plasma exchange (7). It should be added that treatment of irAEs with immunosuppressive agents does not appear to affect the antitumor activity of ICIs (13).

## 3 Development of immune checkpoint inhibitors-associated acute kidney injury

### 3.1 Definition, grading, classification of AKI and ICI-AKI definition

Renal adverse effects caused by ICIs usually manifested as AKI (6). In the current studies (14–16), AKI was defined as a 1.5-fold increase in serum creatinine (SCr) from baseline or an increase of ≥0.3 mg/dL (26.5 µmol/L) after starting ICIs. The Kidney Disease Improving Global Outcomes (KDIGO) criteria were used to grade AKI according to the relative change in SCr (16–18). Similarly, the National Cancer Institute’s Common Terminology Criteria for Adverse Events (NCI-CTCAEs) defines and grades AKI by comparing SCr with an “upper limit of normal” cutoff parameters (19). However, this approach often underestimates the incidence of AKI because it ignores those AKI that increase in the normal range of creatinine (mostly low-grade AKI) (20).

Previous studies have divided AKI into four categories based on its etiology, including ICI-AKI, hemodynamic AKI/acute tubular necrosis (ATN), obstructive AKI and AKI of undetermined cause. Nephrologists attribute the cause of ICI-AKI directly to ICIs. Hemodynamic AKI/ATN refers to AKI that occurs in the context of dehydration (such as circulatory failure, diarrhea, vomiting, etc.), tumor lysis syndrome, sepsis, or ischemic ATN (14, 18, 21).

### 3.2 Possible mechanisms of ICI-AKI

**Figure 2:**
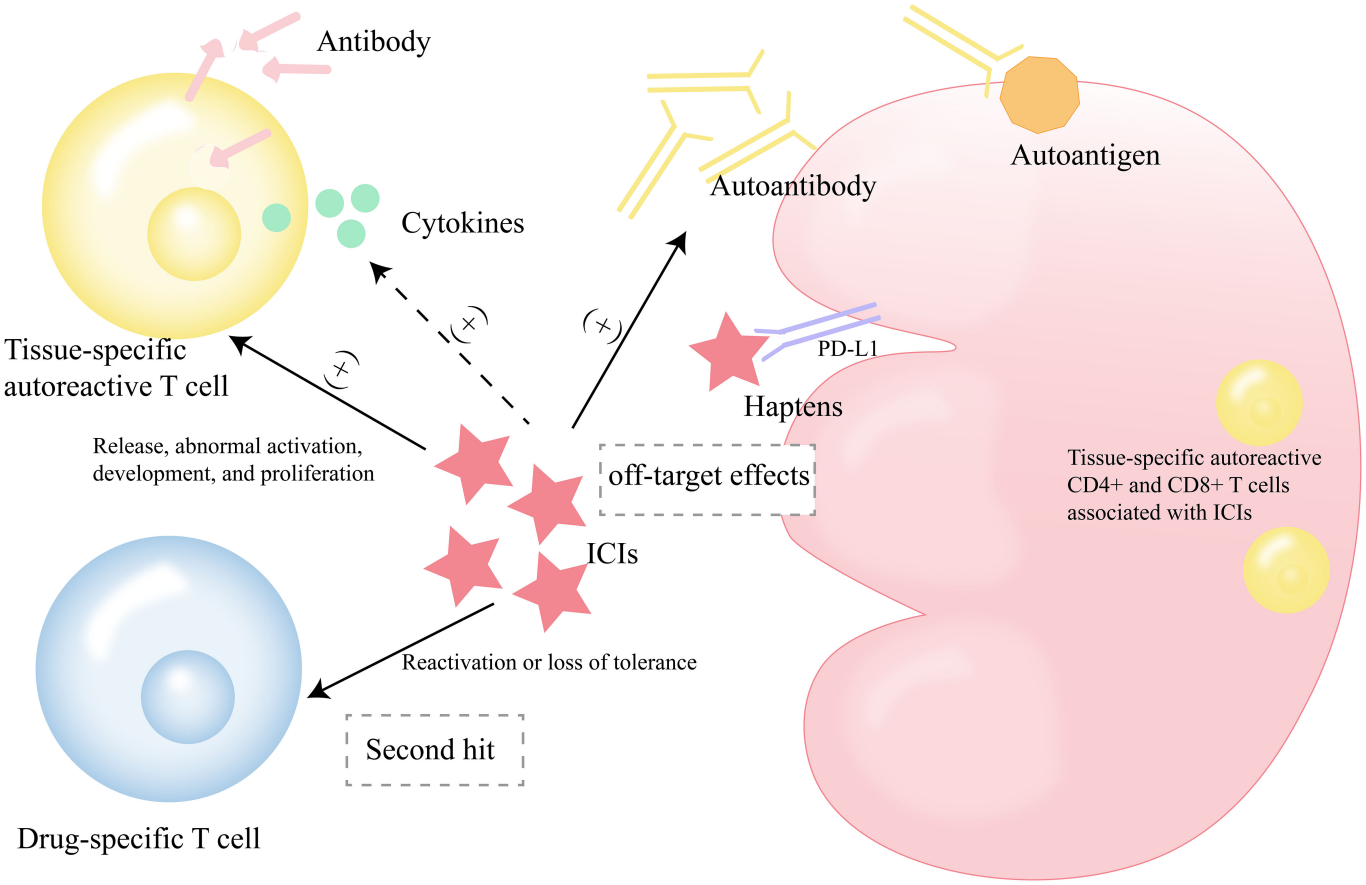
Possible mechanisms of ICI-AKI. ICIs, immune checkpoint inhibitors; ICI-AKI, immune checkpoint inhibitors-associated acute kidney injury.

Different studies have hypothesized the mechanism of ICI-AKI (**Figure 2**). First, ICIs, including anti-CTLA4 and anti-PD-1, may favor the generation of autoantibodies against autoantigens on renal tubular epithelial cells (TECs), mesangial cells, and podocytes, such as anti-dsDNA and antinuclear antigen antibodies (22–25). They engage in an autoimmune reaction with specific renal autoantigens. Some studies have found that the treatment and interruption of ICIs and corticosteroids treatment affect the levels of some autoantibodies in the serum circulation, such as lupus-like glomerulopathy and anti-dsDNA and anti-nuclear antigen antibodies, which are very similar to the phenotype of autoimmune lupus nephritis (22–24, 26).

Second, another mechanism may be that ICIs themselves or bind to kidney-expressed checkpoint receptors to form haptens, known as “off-target effects” (25), which are recognized by local dendritic cells (DCs) and trigger immune responses after acquiring immunogenicity when they are metabolized by renal tubular cells or when they bind to carrier proteins to form antigen-antibody complexes (27, 28). This “reprogramming” of the immune system results in the loss of peripheral tolerance to renal endogenous antigens (29, 30). It has been found that PD-L1 is expressed in renal TECs (31). In a murine model of nephrotoxic nephritis, Neumann et al. found that Foxp3^+^ regulatory T cells (Tregs) infiltrating the kidney express PD-L1. After inhibition of PD-L1 signaling in the murine model (knockout or blockade of PD-L1), Tregs are increased in the kidney, ultimately exacerbating nephrotoxic nephritis. It should be noted that the gene expression profile of Tregs is also altered by the lack of PD-L1 in renal inflammation. Furthermore, Tregs isolated from the PD-L1-depleted murine model were found to have impaired suppressive capacity *in vitro* and no protective effect on nephrotoxic nephritis *in vivo*, indirectly suggesting a protective role of PD-L1 in renal injury (32). In addition, the study by Shim et al. found that PD-1/PD-L1 interaction modulated T cell infiltration and lipocalin 2 expression in kidney transplant murine (33).

It is reasonable to hypothesize that ICIs promote the release, uncontrolled aberrant activation, development, and proliferation of quiescent tissue-specific autoreactive T cells (34, 35). These CD4+ and CD8+ T cells associated with ICIs can be found in different tissues such as myocardium, skeletal muscle, kidney and tumor, and cross-react with these normal tissues (25, 36). They even produce antibodies to mobilize the typical antibody-induced hypersensitivity (37, 38). In addition, activated T cells infiltrate the renal parenchyma and release cytokines to produce an inflammatory response (39). At present, it has been found that ICIs can promote the increase of proinflammatory cytokines/chemokines such as IL-1Ra, CXCL10, TNF-α, IL-6 (25, 40). It has been found that the expression of PD-L1 at both mRNA and protein levels in the proximal tubules in normal kidney tissue is low enough to cause organ-specific immunity (31).

An alternative hypothesis is that ICIs reactivate drug-specific T cells or lose tolerance to them (41). These T cells are produced when an immune response is triggered by prior treatment with drugs known to cause acute interstitial nephritis (AIN) (nonsteroidal anti-inflammatory drugs, proton pump inhibitors, etc.) or other nephrotoxic drugs (antibiotics, etc.) (25, 34, 35, 37). This mechanism can be called the “second hit” (14). To a certain extent, these drugs act as exogenous antigens or haptens as potentiators for the occurrence of ICIs-related irAEs.

### 3.3 Clinical features of ICI-AKI

#### 3.3.1 Incidence for ICI-AKI

Nephrotoxicity associated with ICIs is uncommon compared with other common irAEs, such as dermatitis, enterocolitis, and thyroiditis (42, 43). A review of irAEs with ipilimumab summarized rates of skin toxicity from 47% to 68%, diarrhea from 44%, hypophysitis from 1% to 6%, and hepatotoxicity from 3% to 9% (44). A review of endocrine dysfunction published by Barroso-Sousa et al. (45) concluded that in combination therapy with nivolumab plus ipilimumab, the incidence of hypothyroidism, hyperthyroidism, hypophysitis, and autoimmune adrenalitis was 13.2%, 8.0%, 8.0%, and 4.2%, respectively. In a review of management of endocrine and metabolic toxicity published by Spagnolo et al. (46), it was found that the incidence of thyroid disorders was as high as 15-20% and pituitary disorders as high as 10% with the combination of ICIs. In a retrospective study of 740 melanoma patients, 7.3% had serious infections, most of which were bacterial (85%) (47). In a study of 167 patients with malignancies treated with nivolumab, 19.2% developed infectious disease, the most prevalent type of infection was pneumonia, and 78.1% were bacterial (48, 49). However, the estimated incidence of AKI in patients treated with ICIs was found to be 9.9% to 29% and may be rising (25, 34, 50). The different inclusion criteria for AKI in different studies contributed to the differences in incidence. In a retrospective study of 252 patients with malignancies treated with ICIs, the incidence of AKI was 17.9% (51). In a meta-analysis of eighteen articles comprising 12,111 patients receiving ICIs, the incidence of AKI was 16% (6). In a meta-analysis of 48 clinical trials receiving anti-PD-1, the incidence of AKI was 2.2% (52). In a single-center study of 1016 patients with malignancies, the incidence of sustained AKI that lasted at least three consecutive days was 8% (14).

A summary of previous studies found that the incidence of ICI-AKI ranged from 2.2% to 7.1%, which was lower than the incidence of AKI (14, 16, 52–54). However, delayed onset of AKI after initiation of ICIs or the presence of mild AKI that can be easily attributed to other etiologies may lead to an underestimation of the true incidence of ICI-AKI (55). In a study of 239 anti-PD-1 treated patients with advanced melanoma, AKI was mostly pre-renal (49%). This may be related to gastrointestinal irAEs (diarrhea and colitis) and the use of renin-angiotensin-aldosterone system inhibitors (RAASi), whereas ICI-AKI accounted for only 3.3% (18). In addition, AKI in patients treated with ICIs, including ICI-AKI, was mostly low-grade (14, 18, 54, 55). This is consistent with the conclusion that the incidence of grade 3-4 irAEs is lower than that of low-grade irAEs after treatment with ICIs alone or in combination (56). In the study of 676 patients treated with ICIs, the incidence of stage 1 AKI was 76%, and the incidence of stage 1 ICI-AKI was 81.3% (55). However, in a case-control study, only 77 (17.9%) of 429 patients with ICI-AKI had stage 1, while 48.5% had stage 3, which may be related to the diagnosis and inclusion criteria of ICI-AKI (57).

After establishing that ICIs treatment is associated with a higher risk of AKI compared with non-nephrotoxic drug treatment (52, 58), we also found that the incidence of AKI varies according to ICIs treatment. The risk for ICI-AKI was found to be lower with anti-PD-L1 than with anti-PD-1 (59), and sustained AKI occurs more frequently with anti-CTLA-4 therapy (14). In a study of the comparative risk of AKI after ICIs treatment published by Liu et al. (59), it was proposed that the risk of AKI was higher with anti-CTLA-4 monotherapy than with anti-PD-1, for example, the risk of AKI grades 1-5 and 3-5 was higher with ipilimumab than with durvalumab. This may be because CTLA-4 signals play a role in the early stage of T cell activation, while the effect of PD-1 pathway in limiting T cell activity occurs later (60), and this upstream action of anti-CTLA-4 and its low specificity make it more toxic than anti-PD-1 (59). In addition, it also suggested that the risk of AKI is higher with combination therapy (anti-CTLA-4 plus anti-PD-1) than with ICIs alone, and ICIs are time-dependent and dose-dependent, that is, the higher the target concentration, the more side effects. These conclusions were confirmed in other studies (29, 61–64). Based on these findings, it seems reasonable that 1 mg/kg nivolumab plus 3 mg/kg ipilimumab (N1I3), ipilimumab (anti-CTLA-4), and tremelimumab (anti-CTLA-4) are the three highest risk regimens (59). Finally, this study also illustrates that RCC and urothelial carcinoma have a significantly higher risk of AKI compared with other tumors (59). One study has given the explanation that RCC has a higher presence of immune cells infiltration compared with other tumors, and therefore has a stronger immune adverse reaction after ICIs treatment (65).

#### 3.3.2 Latency period between ICIs initiation and ICI-AKI

Compared with extrarenal irAEs, there is a longer latency between ICIs initiation and ICI-AKI development. As examples, the common dermatitis usually develops within 4 weeks (44, 66, 67) and colitis within 6 weeks after ICIs treatment (43). Neurological symptoms of immune-related hypophysitis appeared as early as 6 weeks after ICIs treatment, whereas cutaneous toxicity was observed after an average of 3.6 weeks (44). The aforementioned infections developed over an average of 135 days, with 79.6% of infections occurring within the first 6 months after ICIs treatment (47). In contrast, ICI-AKI usually occurs later and the median time to onset varies widely among studies. In this review, we summarized the median time to onset of ICI-AKI ranging from 1 month to 10 months or even longer (34, 53, 68, 69). It may be related to the long half-life of ICIs (70) and the delay of clinical recognition due to the insensitivity of SCr as a marker of kidney injury (62). From the clinical pharmacokinetics analysis, it was found that the prolonged half-life of ICIs was due to their binding to the protective neonatal Fc receptors to avoid lysosomal degradation (70, 71). It should be noted that the type of ICIs also affected the development of ICI-AKI. Some studies have found that ICI-AKI occurs later after anti-PD-1 treatment than anti-CTLA-4 treatment (34). In an analysis of 1918 reports of ICI-related AKI events, durvalumab had the longest time to onset, followed by nivolumab and avelumab (72).

#### 3.3.3 Risk factors for development of ICI-AKI

Previous studies have found that combination therapy with anti-CTLA-4 and anti-PD-1/anti-PD-L1 is associated with a higher risk of ICI-AKI, which seems plausible (25, 68, 73, 74). In the discussion of a case report, it is proposed that combination therapy with ICIs may play a greater role in enhancing antigen recognition and T-cell proliferation in lymph nodes, as well as an unconstrained cytotoxic T-cell effect in peripheral normal tissues, that is, synergism between different ICIs (75). Secondly, a study has found that the addition of chemotherapy to ICIs treatment increased the risk of AKI (59). Interestingly, cumulative dose of anti-PD-1 was found to be an independent risk factor for the development of AKI in a study of anti-PD-1 in advanced melanoma (18). In particular, a systematic review and meta-analysis of 27 studies and a systematic review of 127 studies both found that older age increased the risk of AKI after ICIs treatment (74, 76). Alternatively, in the study by Qu et al. (77), males were found to be at high risk for ICI-related nephrotoxicity. This may be due to male hormones, which increased oxidative stress, activated the renin-angiotensin system, and worsened fibrosis in damaged kidneys (78). In the study of 676 patients treated with ICIs, a gynecologic malignancy was also found to be independently associated with AKI (55).

In addition, the history of some comorbidities in cancer patients is also associated with the development of ICI-AKI. In this review, we summarized that hypertension (53), diabetes (51), hypoproteinemia (serum albumin <30g/L) (21, 51), anemia (21), autoimmune diseases, extrarenal irAEs (18, 57, 74, 79), and chronic kidney disease (CKD) (18, 51, 74) are all associated with the higher risk of ICI-AKI. Theoretically, patients with concurrent extrarenal irAEs are more likely to develop renal adverse effects. Because these patients are more likely to produce overactivated T cells to fight against autoantigens and induce autoimmune responses (6, 68). What’s interesting is that lower baseline estimated glomerular filtration rate (eGFR) is also associated with a higher risk of AKI in some studies (57, 68), while the opposite conclusion has been shown in others studies (14, 53, 80, 81). One explanation is that baseline eGFR may be influenced by other confounding factors, such as age and cardiovascular disease (82). An alternative explanation is that patients with lower baseline eGFR have poorer renal reserve, which would raise the SCr threshold rather than increase the risk of immune damage to ICIs (68).

The combined use of nonsteroidal anti-inflammatory drugs (NSAIDs) (14, 74), antibiotics (83), vitamin K antagonist fluindione (54, 74), RAASi (18, 51) and diuretics (55, 74) in cancer patients will increase the risk of ICI-AKI (21). In addition, acid inhibitors, including proton pump inhibitors (PPIs) (14, 57, 76) and histamine H2-receptor antagonists (H2RAs), reduce the therapeutic effect of ICIs and increase the risk of AKI (84). In the study by Chen et al., although the use of PPIs and NSAIDs were both risk factors for all-cause AKI and ICI-AKI, the odds ratio (OR) was higher for ICI-AKI than for all-cause AKI (1.77 vs. 2.42, 1.77 vs. 2.57, respectively) (85). As mentioned above, studies found that retreatment with ICIs after exposure to drugs known to cause AIN, including PPIs, NSAIDs, and antibiotics, activated drug-specific T cells, leading to loss of tolerance (14, 57). Studies have found that PPIs reduce the efficacy of ICIs by inducing changes in gut microbiota (86) and pH of tissues around tumors, and even directly acting on the immune system in the vicinity of tumor tissues (87, 88). In a case report published by Koda et al. (30), a NSCLC patient treated with lansoprazole developed acute tubulointerstitial nephritis (ATIN) after nivolumab treatment. It is possible that nivolumab altered the long-term tolerability of lansoprazole. This illustrates the importance of stopping those drugs known to induce ATIN, such as lansoprazole, during ICIs treatment. For a better understanding, we summarized the current information on risk factors for incident ICI-AKI in **Table 1**.

**Table 1:** Risk factors for incident ICI-AKI.

Risk factors	Author (reference)	N=	OR/HR^a^	95% CI^a^
combination ICIs therapy	Cortazar et al. (68)	138 (ICI-AKI) 276 (control)	3.88	2.21 to 6.81
	Abdelrahim et al. (73)	1164	3.725, 6.305; 5.101, 9.041.	1.144 to 12.134, 2.436 to 16.318; 1.554 to 16.745, 3.246 to 25.177.
	Caihong Liu et al. (74)	27 studies	2.45	1.40 to 4.31
cumulated doses of anti-PD-1	Stein et al. (18)	239	NA	NA
ipilimumab	Abdelrahim et al. (73)	1164	3.281; 4.096	1.213 to 8.873; 1.415 to 11.856
	Caihong Liu et al. (74)	27 studies	2.66	1.42 to 4.98
the addition of chemotherapy	Fei Liu et al. (59)	85 randomized trials	NA	NA
older age	Caihong Liu et al. (74)	27 studies	1.01	1.00 to 1.03
	Xu et al. (76)	127 studies	1.055	1.016 to 1.097
Comorbidities				
hypertension	Meraz-Muñoz et al. (53)	309	2.96	1.33 to 6.59
diabetes	Guven et al. (51)	252	2.042	0.923 to 4.518
hypoproteinemia			2.848	1.225 to 6.621
	Ji et al. (21)	1615	1.62	1.17 to 2.23
anemia			1.95	1.16 to 3.28
CKD	Caihong Liu et al. (74)	27 studies	2.90	1.65 to 5.11
	Stein et al. (18)	239	NA	NA
	Guven et al. (51)	252	3.385	1.510 to 7.588
Cancer stype				
RCC and urothelial carcinoma	Fei Liu et al. (59)	85 randomized trials	NA	NA
a gynecologic malignancy	Koks et al. (55)	676	3.91	1.55 to 9.85
Concomitant drugs				
PPIs	Cortazar et al. (68)	138 (ICI-AKI) 276 (control)	2.85	1.81 to 4.48
	Abdelrahim et al. (73)	1164	2.387; 2.355	1.328 to 4.291; 1.393 to 3.983
	Caihong Liu et al. (74)	27 studies	2.23	1.88 to 2.64
	Okamoto et al. (84)	11 papers	2.10	1.74 to 2.53
H2RAs				
NSAIDs	Caihong Liu et al. (74)	27 studies	2.61	1.90 to 3.57
vitamin K antagonist fluindione			6.48	2.72 to 15.46
	Espi Liu et al. (54)	352	6.40	1.42 to 26.08
diuretic	Caihong Liu et al. (74)	27 studies	1.78	1.32 to 2.40
	Koks et al. (55)	676	2.61	1.21 to 5.60
RAASi (ACEIs/ARBs)	Caihong Liu et al. (74)	27 studies	1.76	1.15 to 2.68
	Guven et al. (51)	252	2.236	1.017 to 4.919
antibiotics	Seethapathy et al. (83)	599	NA	NA
lower baseline eGFR	Cortazar et al. (68)	138 (ICI-AKI) 276 (control)	1.99	1.43 to 2.76
	Gupta et al. (57)	429(ICI-AKI) 429 (control)	2.23; 2.62	1.35 to 3.68; 1.47 to 4.65.
extrarenal irAEs	Caihong Liu et al. (74)	27 studies	2.34	1.53 to 3.59
	Gupta et al. (57)	429(ICI-AKI) 429 (control)	2.07	1.53 to 2.78

a When the study type is original research, the value is the result of multivariable analysis.

OR, Odds Ratio; HR, Hazard Ratio; CI, Confidence Interval; ICI-AKI, immune checkpoint inhibitors-associated acute kidney injury; ICIs, immune checkpoint inhibitors; PD-1, programmed cell death receptor-1; NA, not available; CKD, chronic kidney disease; RCC, renal cell carcinoma; PPIs, proton pump inhibitors; H2RAs, histamine H2-receptor antagonists; NSAIDs, nonsteroidal anti-inflammatory drugs; RAASi, renin-angiotensin-aldosterone system inhibitors; ACEIs, angiotensin-converting enzyme inhibitors; ARBs, angiotensin-receptor blockers; eGFR, estimated glomerular filtration rate; irAEs, immune-related adverse events.

### 3.4 Outcomes of ICI-AKI

Previous studies have found that more than half of patients with ICI-AKI develop renal recovery, which may be due to the low stage of most ICI-AKI (55, 57). Renal recovery of ICI-AKI after corticosteroid therapy is up to more than 90%. In 5 of 6 case reports of anti-PD-1 associated AIN, renal function returned to baseline after corticosteroid therapy (89). Renal recovery was associated with early corticosteroid use and initial steroid dose. In the study by Manohar et al. (90), from the results of the first month of treatment, in 12 cases of biopsy-proven or clinically suspected ICI-associated AIN (ICI-AIN) treated with steroids, patients with complete recovery received a higher steroid dose than those with partial recovery (median 2.79 [1.45 to 3.2] mg/kg per month versus 1.74 [0.8 to 3.2] mg/kg per month). However, delayed treatment with steroids is associated with worse renal prognosis (57, 91, 92). Correspondingly, higher baseline eGFR (57), higher ICI-AKI grade (stage 2 or 3) (21, 57), and extrarenal irAEs (68) were associated with lower odds of renal recovery.

In contrast, for all-cause AKI, studies found that the mortality rate was as high as 93.2% (21), and the median time to death in patients who developed sustained AKI after ICIs treatment was as short as 22 days (14). However, some studies have demonstrated that AKI is not associated with overall survival (OS) (18, 51, 73) and an increased risk of death (53, 55). This may be related to more renal recovery due to lower AKI grade. On the other hand, tumor progression or related complications such as infections also lead to increased mortality (55). It should be noted that, similar to the risk factors for AKI, some studies have found that prophylactic use of antibiotics (16), use of PPIs (18), lower baseline eGFR (57) and hypoproteinemia (51) are also associated with poor OS. Therefore, the relationship among these risk factors, AKI and OS needs to be further explored in more studies.

### 3.5 Diagnosis of ICI-AKI

#### 3.5.1 Clinical work-up of ICI-AKI

The clinical and laboratory features of ICI-AKI are similar to AKI of other causes and are not sensitive and specific. They include pyuria in about half of patients, microscopic haematuria and subnephrotic proteinuria in a variable proportion of patients, and eosinophilia in a minority of patients (16, 28, 29, 68, 69). In a study of 676 cancer patients treated with ICIs by Koks et al. (55), of 54 AKI patients who underwent urinalysis, 37% had leucocyturia and 33.3% had microscopic hematuria, and of 14 patients with ICI-AKI who underwent urinalysis, they accounted for 42.9% and 21.4%, respectively. In a study by Oleas et al. (93), which included 826 cancer patients treated with ICIs, of the 8 AKI patients who underwent urinalysis, seven patients (87%) had subnephrotic proteinuria, two patients (25%) had microscopic isomorphic haematuria, five patients (62%) had eosinophiluria, and of these 8 patients, one patient (12%) had eosinophilia. In addition, a low fractional excretion of sodium (FeNa) and fractional excretion of urea (FeUrea) suggests prerenal renal injury and may help rule out intrinsic renal injury (94). The difference is that, in contrast to FeNa, FeUrea is not affected by diuretics and thus appears to be more accurate in distinguishing between prerenal and renal AKI (95, 96). Based on the above findings, in addition to regular SCr examination, regular urinalysis and even 24-hour urinary protein quantification may be considered in patients with risk factors for ICI-AKI. Imaging procedures may be an alternative way to exclude other possible causes of AKI, such as an ultrasound to rule out urinary tract obstruction, and A CT scan may be considered after sufficient vigilance for further impairment of the patient’s renal function with intravenous contrast (95, 97). After excluding other causes of AKI, in patients for whom renal biopsy is contraindicated, positron emission tomography-computed tomography (PET-CT) may be useful to further identify ICI-AIN (98). In the study by Qualls et al., PET-CT of patients diagnosed with ICI-AIN showed an increased uptake of ^18^F-flourodeoxyglucose (FDG) in the renal cortices bilaterally. In contrast, for those with non-AIN AKI, including prerenal azotemia or cardiorenal syndrome, FDG uptake after AKI was unchanged or slightly decreased from baseline before AKI. However, FDG uptake is subject to various factors (e.g., PET acquisition protocols, reconstruction algorithms, and differences in the timing and dose of FDG injection). The comparison of FDG uptake after AKI and baseline (before AKI) may be more beneficial to improve the diagnostic clarity (99).

#### 3.5.2 Histopathologic features of ICI-AKI

In the present case reports and series, the most common histopathology of ICI-AKI is ATIN (57, 68, 100). However, glomerular or tubular pathologies have been described in several studies, including thrombotic microangiopathy (TMA) (29, 101), pauci-immune glomerulonephritis (102), membranous glomerulonephritis (MN) (53), minimal change disease (MCD) (103, 104), C3 glomerulonephritis, immunoglobulin A (IgA) nephropathy (105), lupus nephropathy, focal segmental glomerular sclerosis (106), granulomatous formations with multinucleated giant cells, renal tubular acidosis (RTA) (107, 108), and acute tubular injury (ATI) (69). In a study of 16 patients who underwent renal biopsy after ICIs treatment, Mamlouk et al. found that 14 patients had histopathological ATIN, alone or in combination with interstitial inflammation associated with glomerular pathologies, including pauci-immune glomerulonephritis, MN, C3 glomerulonephritis, IgA nephropathy, and amyloid A (AA) amyloidosis (28). In a recent prospective study, 10 patients with ICI-AKI all had AIN, of whom 9 had ATI. However, 4 patients with AKI caused by other causes showed ATI and renal thrombotic microangiopathy, with mild to severe interstitial fibrosis or tubular atrophy (15). In particular, these lesions are often accompanied by infiltration of predominantly CD3+ and CD4+ T lymphocytes, varying degrees of mononuclear cells, eosinophils, and plasma cells, interstitial edema, and granuloma (29, 90).

At present, clinical manifestations and laboratory tests are often not reliable in predicting renal lesions and non-AIN lesions have a poor response to empirical corticosteroids treatment (28). However, Renal biopsy is helpful to confirm the diagnosis, guide the withdrawal of ICIs and the treatment of corticosteroids, and assess the rechallenge of ICIs treatment. Therefore, after joint evaluation by nephrologists and oncologists to rule out contraindications, we recommend renal biopsy in patients with stage 2 or higher AKI (28).

#### 3.5.3 Biomarkers for AKI development after ICIs treatment

With the wide application of ICIs in anti-tumor therapy, according to the existing literature, many biomarkers, including circulating blood cell count, cytokines, autoantibodies, etc., can be used to assist in judging the risk and development of irAEs and evaluating the prognosis of patients. However, the specific biomarkers for AKI development are still limited. At present, some studies have found that IFN-α–induced transcript can be used to distinguish T cell-mediated rejection from ICI-AIN after ICIs treatment (109). Lower soluble CD25 and higher soluble CD163 are associated with higher irAEs and urinary soluble CD163 can also be used to identify the etiology of AKI. To be specific, urinary soluble CD163 levels were significantly higher in glomerulopathy injuries than in interstitial or tubular injuries (110, 111). Furthermore, a study also found urinary soluble CD163 is correlated with histological features of ANCA-associated glomerulonephritis and can be used to recognize relapsing ANCA-associated glomerulonephritis (112). Urine retinol binding protein/urine creatinine (uRBP/Cr) is useful in distinguishing ICI-AKI from other causes of AKI (62). In a cohort study of biomarkers for ICI-AKI, serum C-reactive protein (CRP) and uRBP/Cr measures were higher in patients with ICI-AKI than in patients with non-ICI-AKI (113). Therefore, in our opinion, an increase in these two markers may be considered as an indication for renal biopsy when infectious causes have been ruled out.

It should be noted that studies have shown that IL-17, IL-6 are associated with irAEs. And the importance of anti-IL-17 and anti-IL-6 in enhancing the efficacy of ICIs and controlling the development of irAEs has been confirmed (114, 115). Similarly, there are evidences that modulation of the gut microbiota enhances the antitumor effects of ICIs (116, 117). However, at present, studies on the association of these cytokines and gut microbes with ICI-AKI are very limited. In the future, the biomarkers for AKI development need to be further studied.

### 3.6 Management of ICI-AKI

#### 3.6.1 Treatment of ICI-AKI

**Figure 3:**
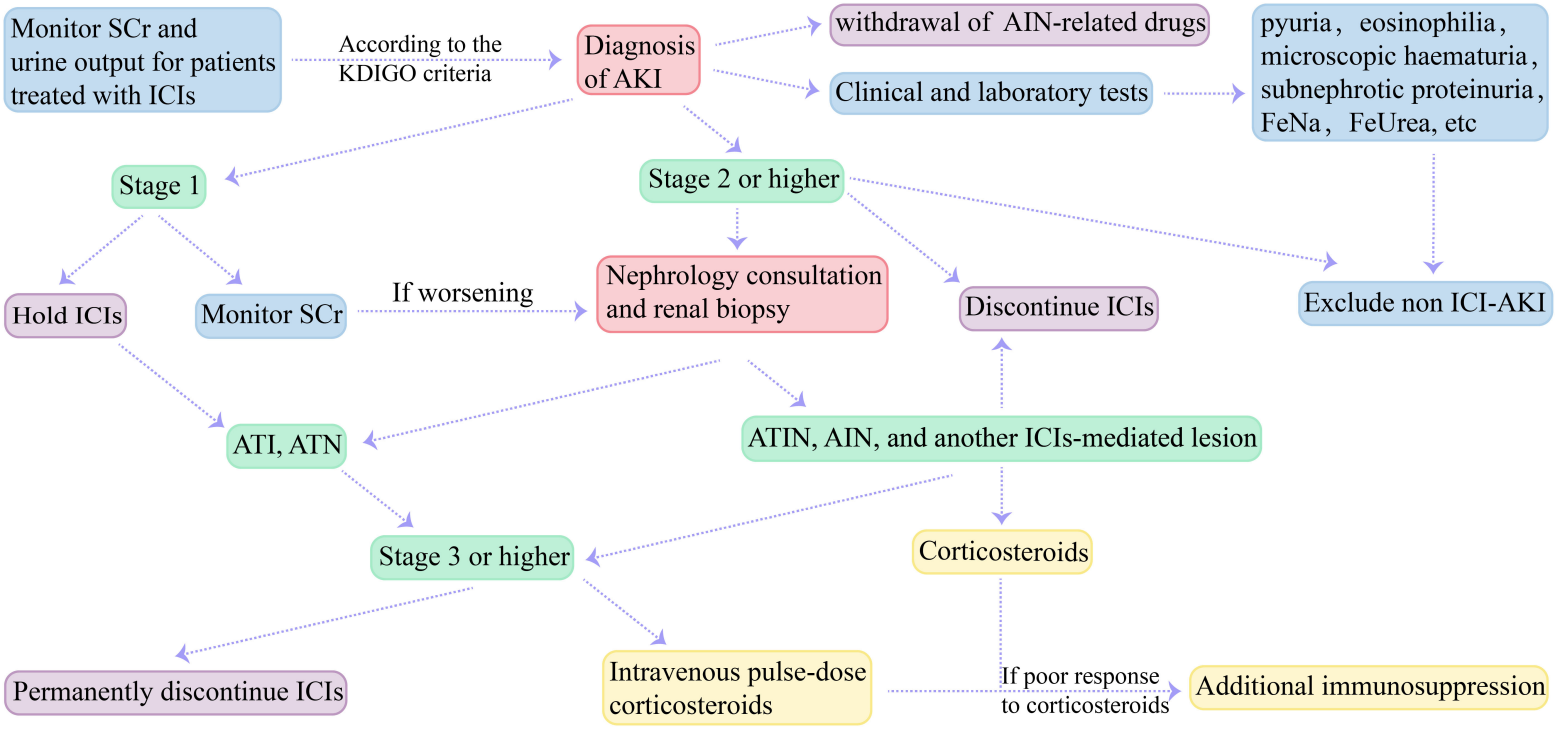
Diagnosis and treatment recommendations for ICI-AKI. ICIs, immune checkpoint inhibitors; SCr, serum creatinine; KDIGO, Kidney Disease Improving Global Outcomes; AKI, acute kidney injury; AIN, acute interstitial nephritis; FeNa, fractional excretion of sodium; FeUrea, fractional excretion of urea; ICI-AKI, immune checkpoint inhibitors-associated acute kidney injury; ATI, acute tubular injury; ATN, acute tubular nephritis; ATIN, acute tubulointerstitial nephritis.

Treatment of ICI-AKI includes withdrawal of ICIs and AIN-related drugs (e.g., PPIs and NSAIDs), and administration of medications such as corticosteroids on the basis of renal biopsy. Previous studies suggested that ICIs should be discontinued in patients with stage 2 or higher ICI-AKI, especially in those without pre-renal AKI or evidence of urinary obstruction (18, 35, 50). Patients with persistent stage 1 or more than stage 2 AKI (including stage 2) should undergo nephrology consultation and consider renal biopsy (118–120). It is recommended to discontinue ICIs when the result is ATIN, AIN, or another immune-mediated lesion, whereas patients with renal biopsy showing ATI or ATN should not discontinue ICIs (121). The Common Terminology Criteria for Adverse Events (CTCAE) grade 3 or higher nephrotoxicity should permanently discontinue ICIs therapy (119, 120). Some studies have found that systemic antibiotics, PPIs and anti-PD-1/anti-PD-L1 combination therapy have poor clinical outcomes (122). Therefore, AIN-related drugs (e.g., PPIs and NSAIDs) should be discontinued in patients with ICI-AKI. Earlier corticosteroid treatment was found to be associated with a better prognosis (57, 91, 92). Therefore, empirical corticosteroids can be used in patients whose causes other than ICIs have been ruled out and who have contraindications to renal biopsy (68). Patients with mild ICI-AKI can only receive oral prednisone (1mg/kg), and patients with stage 3 ICI-AKI or refractory renal dysfunction can receive intravenous pulse-dose corticosteroids (123, 124). In addition, additional immunosuppression including mycophenolate mofetil, infliximab, rituximab, and cyclophosphamide can be used for patients with refractory cases who do not respond well to high-dose corticosteroids (35). A case of mycophenolate mofetil for the treatment of nephrotic syndrome was previously reported (106). Similarly, Lin et al. studied the use of infliximab in 10 patients with ICI-ATIN and found that infliximab was effective in patients who had severe side effects or had a poor response to corticosteroids therapy (125). We summarized recommendations for the diagnosis and treatment of ICI-AKI in **Figure 3**. The recommendations for corticosteroids treatment vary from one guideline to another. The clinical practice guidelines for the management of ICIs-associated nephrotoxicity were summarized in **Table 2** (118–120, 126, 127).

**Table 2:** ASCO, NCCN, SITC, and ESMO clinical practice guidelines for the management of ICIs-associated nephrotoxicity.

Treatment	Stage/Grade ^a^	Guidelines			
		ASCO	NCCN	SITC	ESMO
ICIs interruption	1	NO	NO	NO	NO
	2	NO, but if worsening, YES.		YES	
	3	YES			
	4				
Corticosteroids (prednisone) or additional immunosuppression	1	NO		The first-line treatment for ICI-TIN is glucocorticoids. If ineffectiveness, infliximab or mycophenolate mofetil.	NO
	2	Initial dose of 0.5-1 mg/kg/d, if worsening, increase to 1-2 mg/kg/d.			If worsening, initial oral dose of 0.5-1 mg/kg.
	3	Initial dose of 1-2 mg/kg/d, if worsening, consider additional immunosuppression.			If worsening, initiate intra-venous methyl-prednisolone 1-2 mg/kg.
	4				
ICIs rechallenge	1	YES	If improved to ≤Grade 1, YES.	YES	If improved to ≤Grade 1, YES
	2			NA	
	3	NO			NA
	4				
Others	1	SCr monitoring	SCr and urine protein monitoring	• Concomitant medications known to cause ICI-ATIN interruption • Nephrology consultation • If the lack of specific clinical features of ICI-AKI, renal biopsy.	• Other causes assessment • Other nephrotoxic drugs interruption • Personalized renal biopsy
	2	• Nephrology consultation • Other causes assessment	Nephrology consultation		
	3		Renal biopsy		
	4				

a Nephrotoxicity can be graded using the CTCAE scale and can also be graded with the KDIGO) criteria.

ASCO, American Society of Clinical Oncology; NCCN, National Comprehensive Cancer Network; SITC, Society for Immunotherapy of Cancer; ESMO, European Society for Medical Oncology; ICIs, immune checkpoint inhibitors; ICI-TIN, immune checkpoint inhibitor-associated acute tubulointerstitial nephritis; ICI-AKI, immune checkpoint inhibitor-associated acute kidney injury; NA, not available; SCr, serum creatinine.

#### 3.6.2 ICIs rechallenge after ICI-AKI

Another unsettled question is whether ICIs rechallenge after improvement of renal function in ICI-AKI patients, which is reported by the available literature documenting. In the study by Gupta et al. (57), 28.2% of 429 ICI-AKI patients underwent ICIs rechallenge; of these patients, 76.9% had renal recovery and 16.5% had recurrent ICI-AKI. Fortunately, survival was similar between patients who underwent rechallenge and those who did not. Studies have shown that 16% to 23% of patients with ICI-AKI who receive ICIs rechallenge have a relatively low risk for recurrence (62). Therefore, for the rechallenge of ICIs in patients with ICI-AKI, studies considered that for patients with no ATIN or other ICIs caused lesions on renal biopsy, patients with ATI on renal biopsy, or patients with AIN who have a good response to corticosteroid therapy, when the ICI-AKI has recovered below stage 1 and there is no recurrence, ICIs can be considered. Concomitant low-dose prednisone therapy may be used to reduce the risk of renal irAEs (90, 100, 124).

Finally, studies have shown that ICIs can be safely used in patients with liver or kidney impairment, even in patients with CKD undergoing dialysis treatment, because ICIs are not cleared by the liver or kidney (128–130). The efficacy and safety of ICIs are not affected by age (131). Alternatively, preexisting autoimmune disease is not an absolute contraindication to ICIs treatment (132). Based on the available data and the lifesaving nature of ICIs, it seems to us that the recurrence rate of ICI-AKI after ICIs rechallenge is currently relatively low. For patients with ICIs as the only effective anti-tumor therapy, ICIs rechallenge can be performed after nephrologists and oncologists discuss its benefits and risks together. At the same time, SCr, urine and electrolytes should be monitored, and RRT should be performed when necessary.

## 4 Kidney transplant and ICIs therapy

The treatment of ICIs increases the risk of allograft rejection in graft recipients. This acute rejection is due to the activation of graft-specific T cells (133). In a study of 39 solid organ transplantation recipients treated with ICIs at MD Anderson Cancer Center, 41% had allograft rejection, 81% had graft loss, and 46% died (134). Similarly, in a retrospective cohort study of 69 kidney transplant recipients treated with ICIs, 42% experienced acute rejection, of which 19 experienced graft loss. This rejection was 50% mediated by pure T cells and 50% by a mixture of acute T cells and antibodies (135). In a recent review, it was summarized that 10% to 65% of solid organ transplant recipients treated with ICIs are at risk for acute allograft rejection, with a median onset time of 21 days after treatment initiation, and 24% to 81% of solid organ transplant recipients may lose their allografts (136). The difference is that anti-PD-1/PD-L1 seems to be more likely to cause rejection in kidney transplant recipients than anti-CTLA-4 (34, 137, 138).

However, for kidney transplant recipients in whom ICIs are the only effective antitumor therapy, previous studies have suggested strategies to mitigate the risk of rejection and increase the efficacy of ICIs. First, baseline immunosuppression was maintained in kidney-transplant recipients before treatment with immune checkpoint inhibitors (139). Second, to increase allograft tolerance, mini-pulse steroids may be used concomitantly at the initiation of ICIs (138). Most importantly, switching calcineurin inhibitors (CNI) to mammalian target of rapamycin inhibitors (mTORi) prior to ICIs treatment can improve OS of cancer patients and grafts (140, 141). In turn, dual immunosuppression regimen combining mTORi and corticosteroids or CNI also help maintain immunosuppression (136). In conclusion, ICIs can be used in kidney transplant recipients after weighing the benefits and risks of antineoplastic therapy and graft loss when no other similarly effective treatment options are available. However, possible rejection after ICIs initiation must be alert and prevented as much as possible.

## 5 Electrolyte abnormalities associated with ICIs

Anti-tumor therapy with ICIs can cause a variety of electrolyte abnormalities. Hyponatremia was the most common, and others included hypokalemia, hyperkalemia, hypophosphatemia, and hypocalcemia. In a retrospective observational study of 2458 patients treated with ICIs, hyponatremia occurred in 62%. In terms of severe electrolyte abnormalities, the proportions of hypophosphatemia, hypokalemia and hyponatremia were higher (17%, 6% and 6%, respectively). Hypocalcemia and hyperkalemia accounted for 0.2% and 0.6%, respectively (142). In addition, in a meta-analysis of patients with advanced NSCLC who received ICIs, the incidence of hyponatremia was 8.7% in the study groups of six randomized controlled trials; in 7 randomized controlled trials, the incidence of hypokalemia in the study group was 10.4% (143). The causes of electrolyte abnormalities are diverse. Immune-endocrine disease is an important cause of electrolyte abnormalities, such as hypophysitis, adrenal insufficiency, primary hyperparathyroidism and hypothyroidism (144–146). In the study by Patel et al., the mechanisms of hyponatremia include hypovolemic hyponatremia due to hemodynamic disorders caused by volume depletion, hypervolemic hyponatremia due to CHF or nephrosis, syndrome of inappropriate antidiuretic hormone secretion, and endocrine diseases (147). In addition, Izzedine et al. summarized the etiology of hypercalcemia, including endocrine disorders, sarcoid-like granuloma, humoral hypercalcemia due to parathyroid related hormone and pseudo- or hyperprogressive disease during ICIs therapy (148).

Of note, studies have reported concurrent renal complications in patients who developed electrolyte abnormalities after treatment with ICIs. Balakrishna et al. reported a case of hypokalemia after treatment with nivolumab, with increased SCr and 1+ protein on urinalysis, suggesting concurrent AKI (149). In addition, Herrmann et al. reported three cases of patients treated with ICIs who developed electrolyte disturbances secondary to renal tubular acidosis, one of which was accompanied by hypokalemia, and renal biopsy showed chronic active tubulointerstitial nephritis with moderate arteriosclerosis (108). Similarly, Rai et al. reported a case of nivolumab induced adrenal insufficiency with specific presentation of hypotension and hyponatremia, and interestingly, AKI developed in this patient (150). Although studies on electrolyte abnormalities and nephrotoxicity caused by ICIs are limited, an increasing number of case reports have aroused our attention in this regard. In our view, the monitoring and treatment of ICIs nephrotoxicity also requires more attention to electrolytes, and vice versa. But more research is needed on their relationship.

## 6 Conclusion

With the application of ICIs in clinical anti-tumor therapy, its nephrotoxicity is becoming more and more common, often manifested as AKI. The etiology and histopathology of AKI need to be further clarified. Renal biopsy is essential. However, non-invasive diagnostic methods such as biomarkers and imaging are also being developed. More research is also needed to improve the treatment methods, including AKI graded corticosteroids therapy and additional immunosuppression benefits.

Furthermore, the rechallenge of ICIs is also worthy of attention. Based on the clinical benefits of ICIs and relatively controllable renal complications, for cancer patients for whom ICIs is the only effective treatment, the rechallenge of ICIs with concomitant the management of nephrotoxicity may be a more beneficial way after the collaborative discussion between nephrologists and oncologists. However, as with the use of ICIs in the treatment of kidney transplant recipients, kidney damage should be taken seriously, and further studies are needed to refine treatment strategies. At present, studies on electrolyte abnormalities associated with ICIs are limited, and the association with nephrotoxicity needs to be further studied, which is of great significance for the prevention and treatment of nephrotoxicity.

Finally, with the continued benefits of ICIs in anti-tumor therapy, more attention should be paid to its adverse events in the future researches, especially irAEs with poor prognosis and high mortality. In our view, for each of these irAEs, further studies are needed to clarify their possible mechanisms and identify specific biomarkers to aid diagnosis and standardize effective treatment.

## Author contributions

PZ: Conceptualization, Writing – original draft, Writing – review & editing. YG: Conceptualization, Writing – review & editing. ZK: Conceptualization, Writing – review & editing. JW: Writing – review & editing. SS: Writing – review & editing. WH: Writing – review & editing. JL: Writing – review & editing. ZL: Writing – original draft, Writing – review & editing. RW: Writing – original draft, Writing – review & editing.

## Glossary

**Table:**

ICIs	immune checkpoint inhibitors
AKI	acute kidney injury
ATIN	acute tubulointerstitial nephritis
ICI-AKI	immune checkpoint inhibitors-associated acute kidney injury
PD-1	programmed cell death receptor-1
PD-L1	programmed cell death ligand-1
CTLA-4	cytotoxic T lymphocyte-associated antigen-4
RCC	renal cell carcinoma
NSCLC	non-small cell lung cancer
irAEs	immune-related adverse events
TAAs	tumor-associated antigens
MHC	major histocompatibility complex
APCs	antigen-presenting cells
TCR	T cell receptor
PFS	progression-free survival
OS	overall survival
BMI	body mass index
SCr	serum creatinine
KDIGO	Kidney Disease Improving Global Outcomes
RRT	renal replacement therapy
NCI-CTCAEs	National Cancer Institute’s Common Terminology Criteria for Adverse Events
ATN	acute tubular nephritis
TECs	tubular epithelial cells
dsDNA	double-stranded DNA
DCs	dendritic cells
Tregs	regulatory T cells
IL-1Ra	interleukin-1 receptor antagonist
CXCL	C-X-C motif chemokine ligand
TNF-α	tumor necrosis factor-α
IL	interleukin
mRNA	messenger RNA
AIN	acute interstitial nephritis
RAASi	renin-angiotensin-aldosterone system inhibitors
ACEIs	angiotensin-converting enzyme inhibitors
ARBs	angiotensin-receptor blockers
CKD	chronic kidney disease
eGFR	estimated glomerular filtration rate
NSAIDs	nonsteroidal anti-inflammatory drugs
PPIs	proton pump inhibitors
H2RAs	histamine H2-receptor antagonists
OR	odds ratio
HR	Hazard Ratio
CI	Confidence Interval
ICI-AIN	immune checkpoint inhibitors-associated acute interstitial nephritis
LDH	lactate dehydrogenase
FeNa	fractional excretion of sodium
FeUrea	fractional excretion of urea
CT	computed tomography
PET-CT	positron emission tomography-computed tomography
FDG	^18^F-flourodeoxyglucose
TMA	thrombotic microangiopathy
MN	membranous glomerulonephritis
MCD	minimal change disease
IgA	immunoglobulin A
RTA	renal tubular acidosis
ATI	acute tubular injury
AA	amyloid A
PLR	platelet/lymphocyte ratio
NLR	neutrophil/lymphocyte ratio
CRP	C-reactive protein
uRBP/Cr	urine retinol binding protein/urine creatinine
IFN-α	interferon-α
TGF-β1	transforming growth factor-β1
LIPI	Lung Immune Prognostic Index
ICI-ATIN	immune checkpoint inhibitors-associated acute tubulointerstitial nephritis
ASCO	American Society of Clinical Oncology
CNI	calcineurin inhibitors
mTORi	mammalian target of rapamycin inhibitors
NCCN	National Comprehensive Cancer Network
SITC	Society for Immunotherapy of Cancer
ESMO	European Society for Medical Oncology
NA	not available.
